# Supplementary material for: Acceptance and compliance with micronutrient powder (MNP) among children aged 6–23 months in northern Nigeria
Source: PLOS Glob Public Health. 2022 Oct 17;2(10):e0000961. doi: 10.1371/journal.pgph.0000961 (PMC10022258; doi:10.1371/journal.pgph.0000961)
Supplement: S1 Dataset — (ZIP) [file pgph.0000961.s008.zip › S8_Dataset/Spot Checks.zip!/Spot Checks/03072015_A_SO_ ID_2_SP.pdf]

SPOT CHECK RECORDING FORM

State: ___ Adamawa _____________ LGA: ______ Song __________ Community: __________ Bongo Gari __________

Name of Caregiver (Female): ______ PULENA PWANOGI _________________________

Name of Caregiver (Male):____

Sex of index child: ___ M ________ Age of index child (in months): __15______________

Spot Check Information

1. **Date of MNP distribution to household** (circle option below based on LGA name above)**:**
   - **Kebbi:** Birnin Kebbi & Jega LGA: **June 16**
   - **Kebbi:** Danko-wasagu: **June 17**
   - **Adamawa:** Song, Ganye: **June (18)**
   - **Adamawa:** Guyuk **June 19**

2. **Date of Spot Check Observation: 01 – 07 - 2015**

3. **Number of sachets remaining** (Ask the caregiver to show you and count the specific number of sachets remaining)**:**

   45

4. **Number of sachets that should remain using one MNP sachet per day** (Analyst only)**:**

   48

5. **Number of sachets remaining / number of sachets should be remaining** (Analyst only)**:**

   45/48 = 93.8%

Probes

Question 1. You have **XX** MNP sachets remaining. Explain how easy or difficult it was to use the MNP product on a daily basis for your child 6 – 23 months? (Circle one answer)

Very Difficult **Difficult** **Easy** **Very Easy**

Question 2. Describe any specific challenges that you faced that made the MNP difficult to use on a daily basis?

Question 3. Describe specific aspects of the MNP product that you or your child enjoyed that made it easier to use on a daily basis? The way my Child is now eager to eat food whatever he see he want to eat, and the way MNP make him to be more health and it (MNP) build his body makes me to see it very easy.

Question 4. What questions do you have about the MNP based on your experience using it?
What is that MNP is just for children from 6 – 23 Months?

Question 5. What specific recommendations do you have that we can do as a program to make the MNP easier to use on a daily basis? We have seen changes since from the day that we stake given him MNP, he use to be player not like before he take his mother breast always and whatsoever food he see he will just be eaten and he don't use to be ill like before.
